# Cytolytic activity score as a biomarker for antitumor immunity and clinical outcome in patients with gastric cancer

**DOI:** 10.1002/cam4.3828

**Published:** 2021-03-26

**Authors:** Qingjiang Hu, Kentaro Nonaka, Hiroaki Wakiyama, Yu Miyashita, Yoshiaki Fujimoto, Tomoko Jogo, Kentaro Hokonohara, Ryota Nakanishi, Yuichi Hisamatsu, Koji Ando, Yasue Kimura, Takaaki Masuda, Eiji Oki, Koshi Mimori, Yoshinao Oda, Masaki Mori

**Affiliations:** ^1^ Department of Surgery and Science Graduate School of Medical Sciences Kyushu University Fukuoka Japan; ^2^ Department of Surgery Kyushu University Beppu Hospital Beppu Japan; ^3^ Department of Anatomic Pathology Graduate School of Medical Sciences Kyushu University Fukuoka Japan

**Keywords:** biomarkers, gastric cancer, genomics, immunology, TCGA

## Abstract

**Background:**

A simple measure of immune cytolytic activity (CYT) base on mRNA expression levels of two genes, *GZMA* and *PRF1*, was recently reported. Here, we aimed to evaluate the CYT score's potential as a measure of antitumor immunity and predictor of clinical outcome in gastric cancer (GC) patients.

**Materials and Methods:**

We evaluated the correlations between tumor‐infiltrating immune cells and the CYT score in 238 GC samples from The Cancer Genome Atlas (TCGA). Next, we investigated CYT score associations with molecular subtypes, somatic mutation load, and immune checkpoint molecules in GC samples from TCGA and Asian Cancer Research Group (ACRG). Moreover, we evaluated the clinical significance of the CYT score calculated by reverse transcription (RT)‐quantitative PCR (qPCR) data in 123 GC samples and the association of the CYT score with the response to anti‐PD‐1 therapy in 7 GC samples from Kyushu University Hospital.

**Results:**

The CYT score positively correlated with the proportions of tumor‐infiltrating CD8+ T cells and macrophages and negatively correlated with the proportion of regulatory T cells in GC tissues. A high CYT score was associated with common immune checkpoint molecules, a high mutation, the Epstein–Barr virus subtype, and the microsatellite instability subtype in GC. Moreover, a low CYT score was a poor prognosis factor in patients with GC. Finally, the CYT score was higher in a responder to anti‐PD‐1 therapy compared to nonresponders.

**Conclusion:**

The CYT score reflects antitumor immunity and predicts clinical outcome in GC patients.

## Graphical abstract

High CYT score was associated with tumor‐infiltrating CD8+ T cells, EBV, MSI‐H, high tumor mutation load, and upregulated immune checkpoint molecules in gastric cancer. Moreover, the CYT score was associated with prognosis and response to the anti‐PD‐1 antibody in gastric cancer patients.

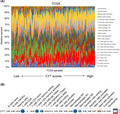

## 1 INTRODUCTION

Gastric cancer (GC) is a common carcinoma with a poor prognosis.^1^ A critical cause of carcinogenesis in GC is the accumulation of somatic mutations, mainly attributed to DNA damage and impaired DNA repair. The strongest known risk factor for GC, *Helicobacter pylori* infection, has been shown to induce DNA damage and DNA repair dysregulation via chronic gastritis.^2^ The accrual of DNA mutations can change the phenotype of cancer cells to favor carcinogenesis and tumor progression. DNA mutations can also produce neoantigens,^3^ which attract and activate immune cells within the tumor microenvironment. Previous studies demonstrated that tumor‐infiltrating lymphocytes (TILs), the majority of which are CD8+ T cells, were involved in the progression of GC and are associated with prognosis.^4^, ^5^, ^6^ A meta‐analysis showed that a high proportion of CD8+ T cells predicted a good prognosis in advanced GC.^7^ Moreover, tumor‐infiltrating macrophages also could predict the prognosis of GC patients.^8^ In contrast, enhanced infiltration of FOXP3+ regulatory T cells was associated with poor prognosis in GC.^9^ Thus, GC biology and the outcomes of GC patients are determined at least in part by tumor‐infiltrating immune cells.

Tumor‐infiltrating CD8+ T cells activation depends on several simultaneous interactions between the T cell and antigen‐presenting cells.^10^, ^11^ When exposed to cancer cells, activated tumor specific CD8+ T cells release the cytotoxin granzymes and perforin. Perforin forms pores on the cancer cell membrane, allowing granzymes to enter the cytoplasm and trigger the caspase cascade to induce apoptosis.^12^ A recent report devised a new index of immune cytolytic activity (CYT), which was calculated using the mRNA expression of perforin (*PRF1*) and granzyme A (*GZMA*).^13^, ^14^

In the past few years, immune checkpoint inhibitors have led to transformative developments in cancer therapy.^15^, ^16^ Previous studies reported that anti‐PD‐1 therapy enhanced the immune response against tumor cells and improved the outcomes of patients with GC.^16^, ^17^, ^18^ Although the combined positive score (CPS),^17^ tumor mutation burden, infection with Epstein–Barr virus (EBV), and microsatellite instability (MSI) correlate with the response to anti‐PD‐1 antibodies in GC,^19^ these predictive biomarkers remain controversial.

The previous study reported that the CYT score was a prognostic biomarker in hepatocellular carcinoma.^20^ The CYT score also correlated with the clinical outcome of colorectal cancer patients.^21^ However, the clinical impact of the CYT score in GC is poorly understood. We hypothesize that the CYT score may reflect antitumor immunity, clinical outcomes, and response to anti‐PD‐1 therapy in GC. Herein, we evaluated the association between the CYT score and tumor‐infiltrating immune cells, mutation load, molecular subtypes, and immune checkpoint molecules in GC. We also clarified the clinical significance of the CYT score in patients with GC.

## 2 MATERIALS AND METHODS

### 2.1 Asian cancer research group

The Asian cancer research group (ACRG) dataset is available in the Gene Expression Omnibus database and consists of clinical data and mRNA expression from 300 GC patients. The mRNA expression data of the 300 GC samples were subjected to quantile normalization. The accession number of ACRG dataset is GSE62254.

### 2.2 The Cancer Genome Atlas

We obtained gene expression data (RNA‐seq) of 238 GC samples, somatic mutation data of 395 GC samples, and clinical assessment data of 223 GC patients in the Firehose pipeline at the Broad Institute. We also obtained the sample IDs of The Cancer Genome Atlas (TCGA) molecular subtypes from TCGA Research Network. The mRNA expression data (RPKM values, raw counts) were subjected to quantile normalization. Total mutation numbers in GC tumor tissues were counted according to somatic mutation data.

### 2.3 Kyushu cohort A

The Ethics and Indications Committee of Kyushu University approved this current study. We obtained tumor tissues from 123 patients with GC. Every participant provided a written informed consent for this study. These participants underwent gastrectomy at Kyushu University Beppu Hospital between 1996 and 2002. There were 24 patients with well‐differentiated adenocarcinoma, 34 patients with moderately differentiated adenocarcinoma, 4 patients with mucinous adenocarcinoma, 47 patients with poorly differentiated adenocarcinoma, and 14 patients with signet ring cell adenocarcinoma. We placed the tumor tissues in RNAlater (Takara) (−80°C).

### 2.4 Kyushu cohort B

The Ethics and Indications Committee of Kyushu University approved this current study. We obtained formalin‐fixed, paraffin‐embedded (FFPE) sections from eight advanced or recurrent GC patients. Every participant provided a written informed consent for this study. These participants had received the anti‐PD1 antibody therapy (nivolumab or pembrolizumab) at Kyushu University Hospital. The FFPE sections were collected at the time of initial diagnosis of GC between 2017 and 2019 before the anti‐PD‐1 antibody therapy. We extracted total RNA from the FFPE sections of eight samples. One sample was excluded from the initial group because of the poor quality of samples. Therefore, we successfully enrolled seven GC patients who had received anti‐PD1 antibody therapy in this study. There were four patients with recurrent and three patients with advanced GC. Response to anti‐PD‐1 therapy was assessed according to the RECIST guidelines (version 1.1).

### 2.5 CYT score

We calculated the CYT score using the geometric mean of *GZMA* and *PRF1* mRNA expression in TCGA dataset, the ACRG dataset, Kyushu cohort A, and Kyushu cohort B, as previously described.^13^

### 2.6 Analysis of CIBERSORT

CIBERSORT (https://cibersortx.stanford.edu) is an online tool to calculate the proportions of immune cells in a mixed cell population using RNA‐seq data. RNA‐seq data of 238 GC samples collected from TCGA were used for the CIBERSORT analysis.

### 2.7 Total RNA extraction

We extracted total RNA from frozen tissues and FFPE sections with ISOGEN (NIPPON GENE).

### 2.8 Reverse transcription and quantitative PCR

We performed reverse transcription using M‐MLV Reverse Transcriptase, as described previously.^22^ Next, we performed quantitative PCR (qPCR) with LightCycler 480 (Roche, Swiss) and SYBR Green I Master Mix. The primers used in this study were listed as follows: *GZMA*, 5′‐CAGTTGTCGTTTCTCTCCTGC‐3′ (F) and 5′‐TGCAGTCAACACCCAGTCTTT‐3′ (R); *PRF1*, 5′‐AATGTGCATGTGTCTGTGGC‐3′ (F) and 5′‐GGGAGTGTGTACCACATGGA‐3′ (R); *GAPDH*, 5′‐AGCCACATCGCTCAGACAC‐3′ (F) and 5′‐GCCCAATACGACCAAATCC‐3′ (R). The mRNA levels of *GZMA* and *PRF1* were normalized to that of *GAPDH*.

### 2.9 Immunohistochemical analysis

We performed immunohistochemical analysis (IHC) analysis of PD‐L1 expression with FFPE sections, as previously described.^22^ The FFPE sections were obtained from GC patients. The monoclonal antibody against PD‐L1 (rabbit) (13684, CST) was used at a dilution of 1:200. An experienced research pathologist of Kyushu University calculated the CPS of PD‐L1, as previously reported.^17^

### 2.10 Statistical analysis

We divided clinical cases into two groups (CYT‐high and CYT‐low) based on CYT scores with the minimum *p*‐value approach, as previously described.^23^ Comparisons between variables were assessed using the Chi‐squared test, Student's *t* test, and Mann–Whitney *U* test, where appropriate. We also used Pearson's correlation coefficient, Kaplan–Meier method, Log–rank test, and Cox regression model in this study. Significance was defined as a two‐sided *p* value < 0.05. JMP 14 software (SAS) and R software version 3.3.2 were used in this study.

## 3 RESULTS

### 3.1 The immune microenvironment in GC

**FIGURE 1:**
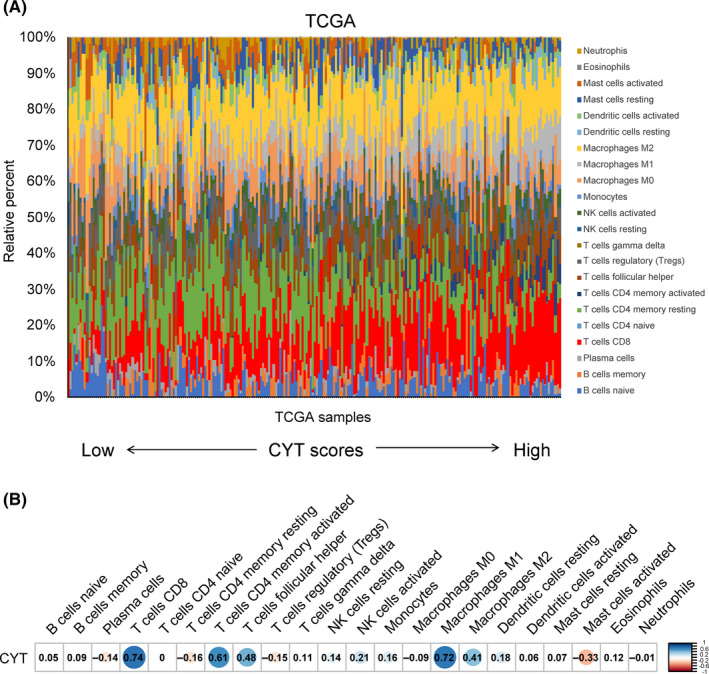
The immune microenvironment and cytolytic activity (CYT) score in gastric cancer (GC) tissues. (A) The proportions of immune cells in each GC samples (The Cancer Genome Atlas [TCGA], *n* = 238) were shown in different colors in the bar chart. The lengths of each bar represented the levels of the immune cell populations. The GC samples were sorted in ascending order of CYT scores. (B) The correlations of immune cells subpopulations with CYT score in GC samples (TCGA, *n* = 238). The values in each box represented the Pearson correlation coefficient. Red represented a negative correlation, and blue represented a positive correlation. The darker the color, the higher the correlation was (*p* < 0.05). No color (white) represented no significance (*p* ≥ 0.05).

We analyzed the gene expression data of 238 GC samples using the CIBERSORT algorithm to investigate the distribution of tumor‐infiltrating immune cells in GC tissues. The relative proportions of 22 tumor‐infiltrating immune cells in each GC sample are shown in Figure 1A. We found relatively high percentages of CD8+ T cells, as well as uncommitted (M0), pro‐(M1), and anti‐inflammatory (M2) macrophages in GC tissues. Naïve CD4+ T cells were not present in GC tissues. The proportion of CD8+ T cells positively correlated with that of M1, M2, activated CD4+ memory T cells, and T follicular helper cells (supporting information Figure S1). Conversely, the proportion of CD8+ T cells negatively correlated with activated mast cells, resting CD4+ memory T cells, and regulatory T cells (supporting information Figure S1). These findings are consistent with the current dogma that CD8+ T cells, and macrophages play a pivotal role in antitumor immunity and regulatory T cells suppress CD8+ T cell numbers.

### 3.2 The CYT score correlates with TILs and macrophage infiltration in GC tissues

Next, we investigated the association between the CYT score and the relative proportions of tumor‐infiltrating immune cells in GC tissues. We found that CYT scores positively correlated with the proportions of CD8+ T cells, M1, M2, activated CD4+ memory T cells, and T follicular helper cells (Pearson correlation's *r* 0.41–0.74, *p* < 0.001, Figure 1B and supporting information Figure S2), which contribute to antitumor immunity.^24^, ^25^ CYT scores negatively correlated with activated mast cells, resting CD4+ memory T cells, and regulatory T cells (Pearson correlation's *r* −0.15 to −0.33, *p* < 0.05, Figure 1B and supporting information Figure S2), which mainly suppress antitumor immunity.^24^, ^26^ These data suggest that the CYT score reflects the level of antitumor immunity in GC tissues.

### 3.3 CYT score is associated with different molecular subtypes of GC

**FIGURE 2:**
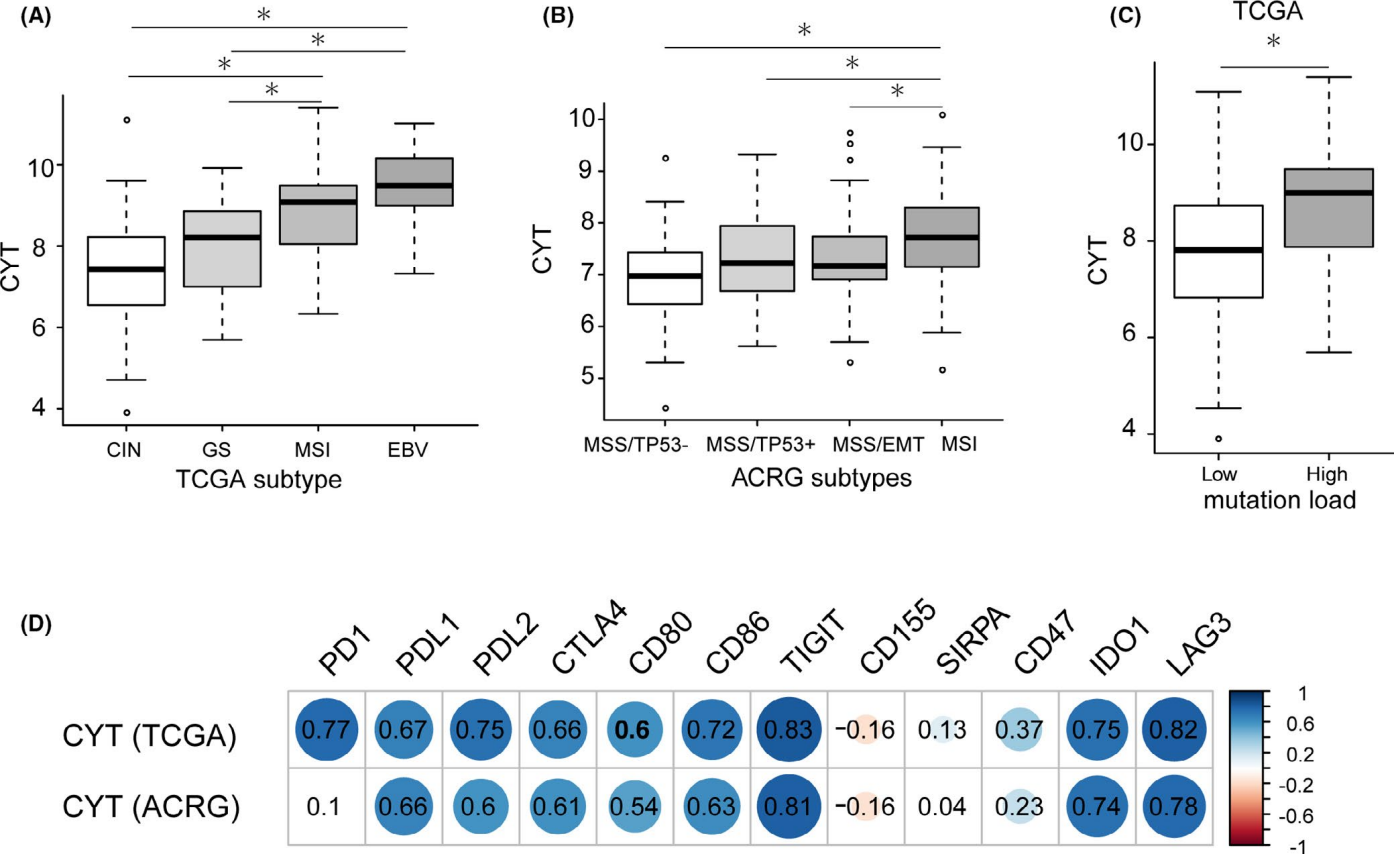
The cytolytic activity (CYT) score associations with molecular subtypes, high mutation load, and immune checkpoint molecules in gastric cancer (GC). (A) CYT score in The Cancer Genome Atlas (TCGA) molecular subtypes. CIN, chromosome instability, *n* = 50; GS, genome stable, *n* = 47; MSI, microsatellite instability, *n* = 107; EBV, Epstein–Barr virus, *n* = 23. *represented Mann–Whitney *U* test *p* < 0.01. (B) CYT score in ACRG molecular subtypes. MSS, microsatellite stable, MSI, microsatellite instability, EMT, epithelial mesenchymal transition. MSS/TP53−: *n* = 107, MSS/TP53+: *n* = 79, MSS/EMT: *n* = 46, MSI: *n* = 68. *represented Mann–Whitney U test *p* < 0.01. (C) CYT score in GC samples with low (*n* = 185) or high (*n* = 51) mutation load from TCGA. Low mutation load was defined as total mutation numbers below 500, and high mutation load was defined as total mutation numbers above 500. *represented Mann–Whitney *U* test *p* < 0.01. (D) The correlations of immune checkpoint molecules with CYT score in GC samples from TCGA (*n* = 238) and Asian Cancer Research Group (ACRG) (*n* = 300), respectively. The values in each box represented the Pearson correlation coefficient. Red represented a negative correlation, and blue represented a positive correlation. The darker the color, the higher the correlation was (*p* < 0.05). No color (white) represented no significance (*p* ≥ 0.05).

Recently, TCGA and ACRG developed two detailed genomic characterizations of GC.^27^, ^28^ TCGA classified GC into four molecular subtypes: EBV‐positive, MSI, genomically stable (GS), and tumors with chromosomal instability (CIN). ACRG also proposed four molecular subtypes of GC based on MSI status, p53, and epithelial‐to‐mesenchymal transition (EMT) gene expression signature. We hypothesized that the CYT score might correlate with different GC molecular subtypes. In TCGA dataset, the CYT score was significantly higher in patients with EBV‐positive and MSI subtypes than in the GS and CIN subtypes (Figure 2A). Similarly, the CYT score in patients with the MSI subtype was significantly higher than that in the other subtypes in the ACRG dataset (Figure 2B). Thus, a high CYT score is associated with the EBV‐positive and MSI subtypes of GC.

### 3.4 A high CYT score is associated with a high mutation load

We also evaluated the association of CYT score with tumor mutation load in GC samples from TCGA dataset. Tumor mutation load was defined by total mutation numbers in tumor tissues of GC. We determined a cut‐off of 500 based on a histogram of total mutation numbers to distinguish GC samples with a high mutation load from those with a low mutation load (supporting information Figure S3). The CYT score in the high mutation load group was higher than that in the low mutation load group (Figure 2C). Thus, a high CYT score is associated with a high mutation load in GC tissues.

### 3.5 CYT score correlated with the expression of immune checkpoint molecules

We also found that CYT scores positively correlated with the expression of common immune checkpoint molecules (PD‐L1, PD‐L2, PD1, CTLA4, CD80, CD86, TIGIT, CD47, IDO1, and LAG3) in GC samples from TCGA dataset (Figure 2D and supporting information Figure S4). Weak correlations between SIRPA and CD155 with the CYT scores were observed, as demonstrated by low correlation efficient values (Figure 2D). We observed similar trends in GC samples from the ACRG dataset (Figure 2D). These data demonstrate enhanced expression of common immune checkpoint molecules in GC samples with a high CYT score.

### 3.6 Prognostic potential of the CYT score in patients with GC

**FIGURE 3:**
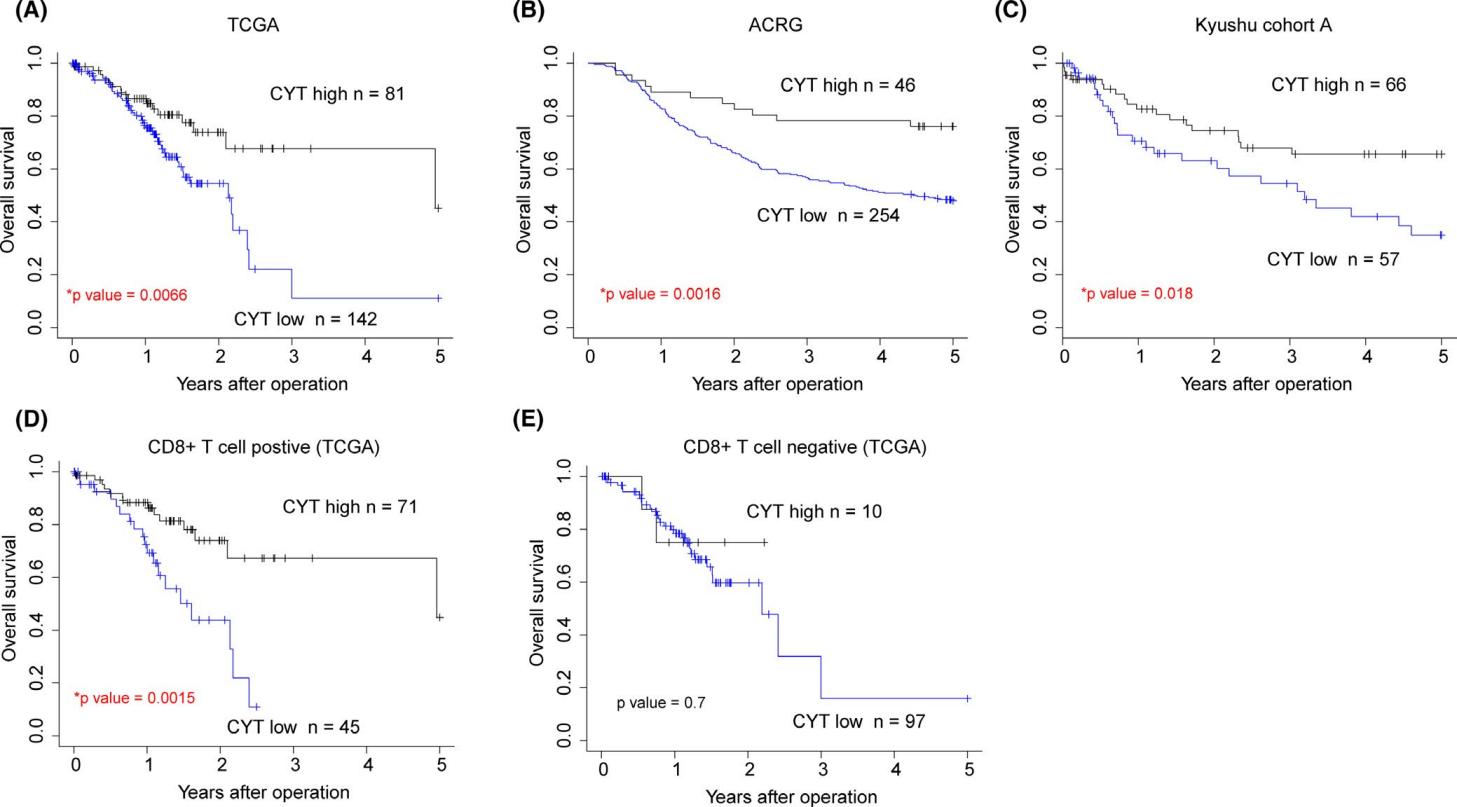
Prognostic significance of cytolytic activity (CYT) score in gastric cancer (GC). (A) The overall survival (OS) of GC patients from The Cancer Genome Atlas (TCGA) (*n* = 223) was analyzed with the Kaplan–Meier method based on the CYT score level. (B) The OS of GC patients from ACRG (*n* = 300) was analyzed with the Kaplan–Meier method based on the CYT score level. (C) The OS of GC patients from the Kyushu cohort A (*n* = 123) was analyzed with the Kaplan–Meier method based on the CYT score level. (D) The OS of 116 GC patients with tumor‐infiltrating CD8 + T cell from TCGA was analyzed with the Kaplan–Meier method. (E) The OS of 107 GC patients without tumor‐infiltrating CD8 + T cell from TCGA was analyzed with the Kaplan–Meier method.

We next evaluated the prognostic potential of the CYT score in patients with GC using three GC cohorts (TCGA, ACRG, and Kyushu cohort A). The GC cases were divided into low and high CYT groups, as previously described.^23^ In all three GC cohorts, patients with low CYT scores exhibited a significantly shorter overall survival (OS) compared to patients with high CYT scores (Figure 3A–C). Importantly, in the Kyushu cohort A, a low CYT score was an independent poor prognostic factor in patients with GC (Table 1).

**TABLE 1:** Univariate and multivariate analyses of clinicopathological factors affecting the overall survival of patients with gastric cancer from the Kyushu cohort A (*n* = 123).

Variable	Univariate analysis		Multivariate analysis	
	Hazard ratio (CI)	*p* value	Hazard ratio (CI)	*p* value
CYT score (low)	2.04	0.018	1.95	0.037
	(1.13–3.79)		(1.04–3.67)	
Depth of tumor invasion (≥SS)	5.91	<0.0001	1.70	0.179
	(2.55–17.16)		(0.78–3.67)	
Lymph node metastasis (+)	8.62	<0.0001	5.39	0.0019
	(3.47–28.78)		(1.86–15.59)	
Lymphatic invasion (+)	4.85	<0.0001		
	(2.21–12.81)			
Vascular invasion (+)	3.40	<0.001		
	(1.82–6.22)			
Tumor size (≥3 cm)	2.70	0.012		
	(1.23–7.11)			
Distant metastasis (+)	9.84	<0.0001	6.32	<0.001
	(3.94–22.36)		(2.40–16.65)	
Peritoneal dissemination (+)	7.61	<0.0001	3.70	<0.001
	(3.84–14.55)		(1.72–7.98)	
Histological type (signet or poorly differentiated adenocarcinoma)	1.33	<0.0001		
	(0.73–2.43)			
Age (≥65 years)	0.65	0.15		
	(0.35–1.17)			
Gender (male)	1.32	0.02		
	(0.70–2.67)			

Abbreviations: CYT, cytolytic activity; SS, subserosa.

We classified patients in TCGA dataset as tumor‐infiltrating CD8+ T cell positive if the relative proportion of CD8+ T cells was higher than the median value. Interestingly, a low CYT score was also associated with poor prognosis in tumor‐infiltrating CD8+ T cell positive patients with GC (Figure 3D). However, there was no significant difference in OS between tumor‐infiltrating CD8+ T cell negative patients with high and low CYT scores (Figure 3E). These data indicated that the prognostic impact of the CYT score is dependent on the proportion of tumor‐infiltrating CD8+ T cells.

### 3.7 Clinicopathological significance of the CYT score in patients with GC

We evaluated the clinicopathological features of patients with GC with a high versus low CYT score using the Kyushu cohort A dataset. There were no significant differences in gender, age, histological type, lymphatic invasion, vascular invasion, tumor size, depth of tumor invasion, distant metastasis, lymph node metastasis, and peritoneal dissemination of patients with a high CYT score compared to those with a low CYT score (Table 2).

**TABLE 2:** The associations between CYT score and clinicopathological factors in gastric cancer patients from the Kyushu cohort A (*n* = 123).

Factors	High CYT score	Low CYT score	*P* value
	(*n* = 66)	(*n* = 57)	
Age (≥65 years)	42 (63.6%)	38 (66.7%)	0.73
Gender (male)	37 (56.1%)	29 (52.7%)	0.71
Histological type			
Well	10 (15.2%)	14 (24.6%)	0.14
Mod	17 (25.8%)	17 (29.8%)	
Muc	4 (6.0%)	0 (0%)	
Por	29 (44.0%)	18 (31.6%)	
Sig	6 (9.0%)	8 (14.0%)	
Depth of tumor invasion (≥SS)	41 (62.1%)	37 (64.9%)	0.75
Lymph node metastasis (+)	40 (60.6%)	37 (64.9%)	0.62
Lymphatic invasion (+)	37 (56.0%)	37 (64.9%)	0.32
Vascular invasion (+)	13 (19.7%)	17 (29.8%)	0.19
Tumor size (≥3 cm)	51 (77.3%)	43 (75.4%)	0.81
Peritoneal dissemination (+)	10 (15.2%)	9 (15.8%)	0.92
Distant metastasis (+)	7 (10.6%)	5 (8.8%)	0.73

Abbreviations: CYT, cytolytic activity; Mod, moderately differentiated adenocarcinoma; Muc, mucinous adenocarcinoma; Por, poorly differentiated adenocarcinoma; Sig, signet ring cell adenocarcinoma; SS, subserosa; Well, well‐differentiated adenocarcinoma.

### 3.8 Association of a high CYT score with an anti‐PD‐1 therapy responder

**FIGURE 4:**
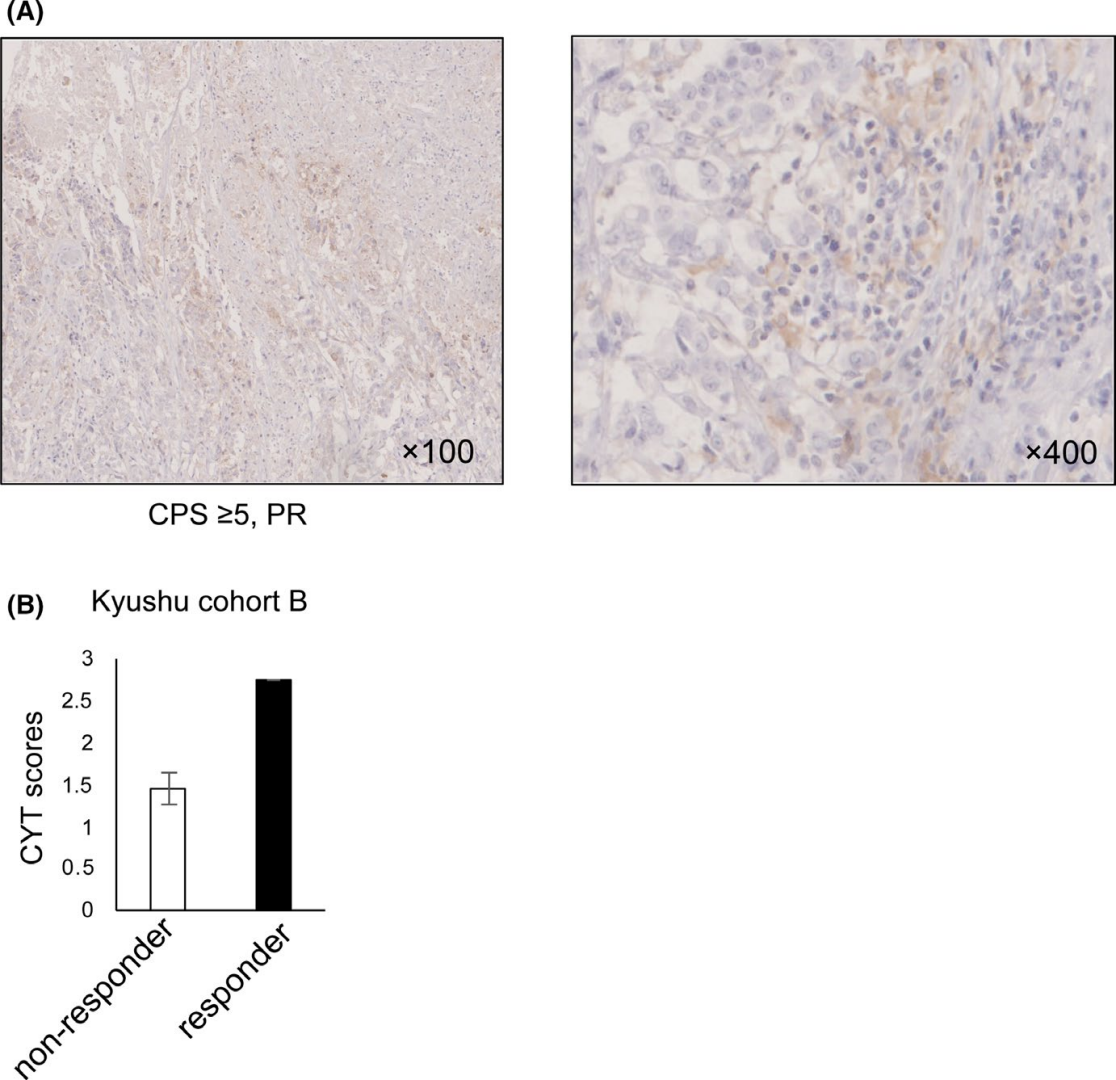
The association between the cytolytic activity (CYT) score and the anti‐PD‐1 antibody response in gastric cancer (GC). (A) The protein level and distribution of PD‐L1 in a responder to the anti‐PD‐1 antibody were shown by analysis of immunohistochemical analysis (IHC). CPS and the anti‐PD‐1 antibody response were shown under the image. CPS, combined positive score. Original magnification, ×100 (left) and ×400 (right). (B) The CYT score in GC patients with PR (*n* = 1, responder) and other responses (*n* = 6, nonresponder) from the Kyushu cohort B. PD, progressive disease, SD, stable disease, PR, partial response, CR, completed response. Other responses consisted of one SD, two non‐CR/non‐PD, and three PD.

Finally, we investigated the relationship between the CYT score and the response to anti‐PD‐1 therapy. We enrolled seven patients with GC, who had received anti‐PD‐1 therapy in the Kyushu cohort B (supporting information Table S1). Overall, the best response to anti‐PD‐1 therapy we observed was partial response (PR) in one case. In addition, we observed progressive disease in three patients, noncomplete response/nonprogressive disease in two patients, and stable disease in one patient. We determined PD‐L1 protein expression in the seven GC cases using a CPS based on IHC analysis (Figure 4A and supporting information Figure S5). Surprisingly, the CPS of PD‐L1 was not associated with the anti‐PD‐1 therapy response (supporting information Table S1). However, the patient who reached PR had a higher CYT score than the other GC cases (Figure 4B), suggesting that the CYT score might be associated with the response to anti‐PD‐1 therapy in GC.

## 4 DISCUSSION

Antitumor immunity is associated with the biology and clinical outcome of several cancers, including GC.^24^ It is widely accepted that tumor‐infiltrating CD8+ T cells are the primary effector cells in the antitumor immune response.^29^ Activated CD8+ T cells, also called cytotoxic T cells, can directly kill tumor cells by releasing granzymes and perforin, which are dramatically upregulated upon activation.^14^ Therefore, the CYT score based on *GZMA* and *PRF1* expression levels is considered a rational method to reflect the activity of tumor‐infiltrating CD8+ T cells and the antitumor immune response.^13^ In this study, we found that the CYT score reflected antitumor immunity and was associated with the clinical outcome of patients with GC.

We evaluated the immune microenvironment of GC tissues by in silico analysis and found that CYT scores positively correlated with the proportions of tumor‐infiltrating CD8+ T cells and macrophages. In the immune microenvironment, cancer stromal cells, including macrophage and cancer‐associated fibroblast, were involved in the activation of CD8+ T cells through antigen presentation, cytokine release, and T cell migration.^24^, ^30^ Hence, the CYT score significantly correlated with the proportions of cancer stromal cells involved in antitumor immunity. In contrast, CYT scores negatively correlated with the proportion of tumor‐infiltrating regulatory T cells that suppress antitumor immunity. These data indicate that the CYT score reflects the level of antitumor immunity in GC. Moreover, we found that a low CYT score was a poor prognostic factor in three independent GC cohorts. The prognostic impact of the CYT score was dependent on tumor‐infiltrating CD8+ T cells. Collectively, low CYT scores represented weak antitumor immunity, which favored tumor progression, resulting in the poor prognosis of GC patients.

We found that the CYT score was significantly higher in patients with the MSI or EBV‐positive subtypes than those with other molecular subtypes of GC. Several studies showed that the MSI and EBV‐positive subtypes each account for about 9% of GC patients.^31^, ^32^, ^33^ MSI is the condition of genetic hypermutability due to DNA mismatch repair deficiency. In GC patients with MSI, hypermutation promoted tumor infiltration of immune cells and antitumor immunity via increased neoantigen load.^3^, ^34^ Consistent with enhanced antitumor immunity, we observed a high CYT score in patients with the MSI subtype of GC. In addition, studies demonstrate that EBV infection recruits lymphocytes into GC tissues.^35^ Moreover, according to a report of TCGA, PD‐L1/2 expression and in some cases, immune cell signaling activation is increased in patients with EBV‐positive GC.^27^ Thus, EBV infection is likely to favor antitumor immunity, consistent with our finding of high CYT scores in patients with EBV‐positive GC.

We also found that the CYT score was higher in GC patients with a high mutation load than those with a low mutation load. Somatic mutations can generate neoantigens,^3^, ^36^ thereby influencing the antitumor immune response in tumor microenvironments. For this reason, a high mutation load can generate abundant neoantigens, resulting in enhanced antitumor immunity and a high CYT score in GC. Moreover, in tumors with a high CYT score, a strong antitumor immune response can select cancer cells with the potential for immune evasion. As a result, the proportion of cancer cells or stromal cells expressing immune checkpoint molecules, including PD‐L1 and PD‐L2, will increase in these tumor microenvironments. We hypothesize that this is one explanation for why the CYT score positively correlated with the expression of common immune checkpoint molecules in this study.

Recent studies divided solid tumors into two groups, referred to as hot and cold tumors, based on the presence of tumor‐infiltrating CD8+ T cells.^24^, ^37^ Hot tumors are characterized by the infiltration of CD8+ T cells and better response to immunotherapy, while cold tumors without CD8+ T cell infiltration have a poor response to immunotherapy.^25^ Our data demonstrated a strong correlation between the CYT score and the proportion of tumor‐infiltrating CD8+ T cells. Additionally, the CYT score can directly reflect the CYT of CD8+ T cells.^14^ Therefore, CYT‐high GC tumors are considered a hot tumor and may benefit from immunotherapy, such as immune checkpoint inhibitors. Interestingly, the Kyushu cohort B showed that the CYT score was higher in a responder to anti‐PD‐1 therapy than nonresponders. The responder also had a high CPS of PD‐L1 in cancer cells and stromal cells. These data suggest that the responder's tumor might be a hot tumor, and the cells expressing PD‐L1 had been preferentially selected for by antitumor immunity. Thus, the anti‐PD‐1 antibody was effective in this patient.

A limitation of this study is the small size of Kyushu cohort B. Further investigations with a large cohort are needed to determine if the CYT score can predict a patient's response to anti‐PD‐1 therapy. In addition, the CYT score was associated with the prognosis of colorectal cancer and hepatocellular carcinoma.^20^, ^21^ Thus, it is also expected to investigate the usefulness of the CYT score in predicting the response to immunotherapy in other solid cancers.

In conclusion, the CYT score reflected the activity of tumor‐infiltrating CD8+ T cells and antitumor immunity in GC. The CYT score was a prognostic biomarker in GC and might be a candidate biomarker for predicting anti‐PD‐1 therapy response.

## 5 ETHICS STATEMENT

This study was approved by the Ethics and Indications Committee of Kyushu University (Japan). The approval ID is 2019–223.

## CONFLICT OF INTEREST

Eiji Oki received lecture fee from Ono Pham.

## Supporting information

Fig S1

Fig S2

Fig S3

Fig S4

Fig S5

Table S1

Supplementary Material

## Data Availability

The data that support the findings of this study are available in the supporting information of this article.
