# Supplementary figures and images for: Cytolytic activity score as a biomarker for antitumor immunity and clinical outcome in patients with gastric cancer
Source: Cancer Med. 2021 Mar 26;10(9):3129–38. doi: 10.1002/cam4.3828 (PMC8085935; doi:10.1002/cam4.3828)

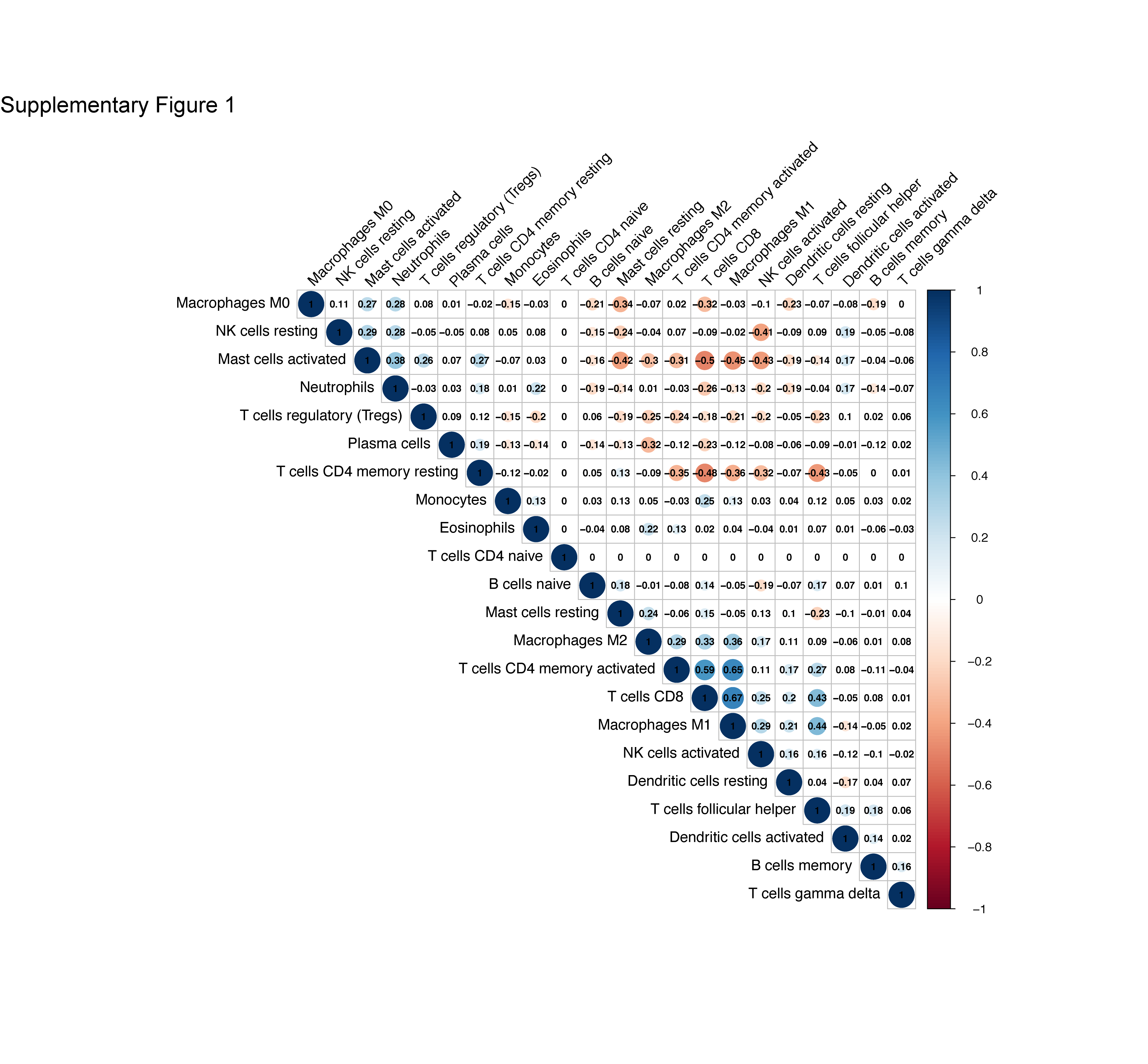

Supplement: Supplementary file 1 — Fig S1 [file CAM4-10-3129-s005.tif]

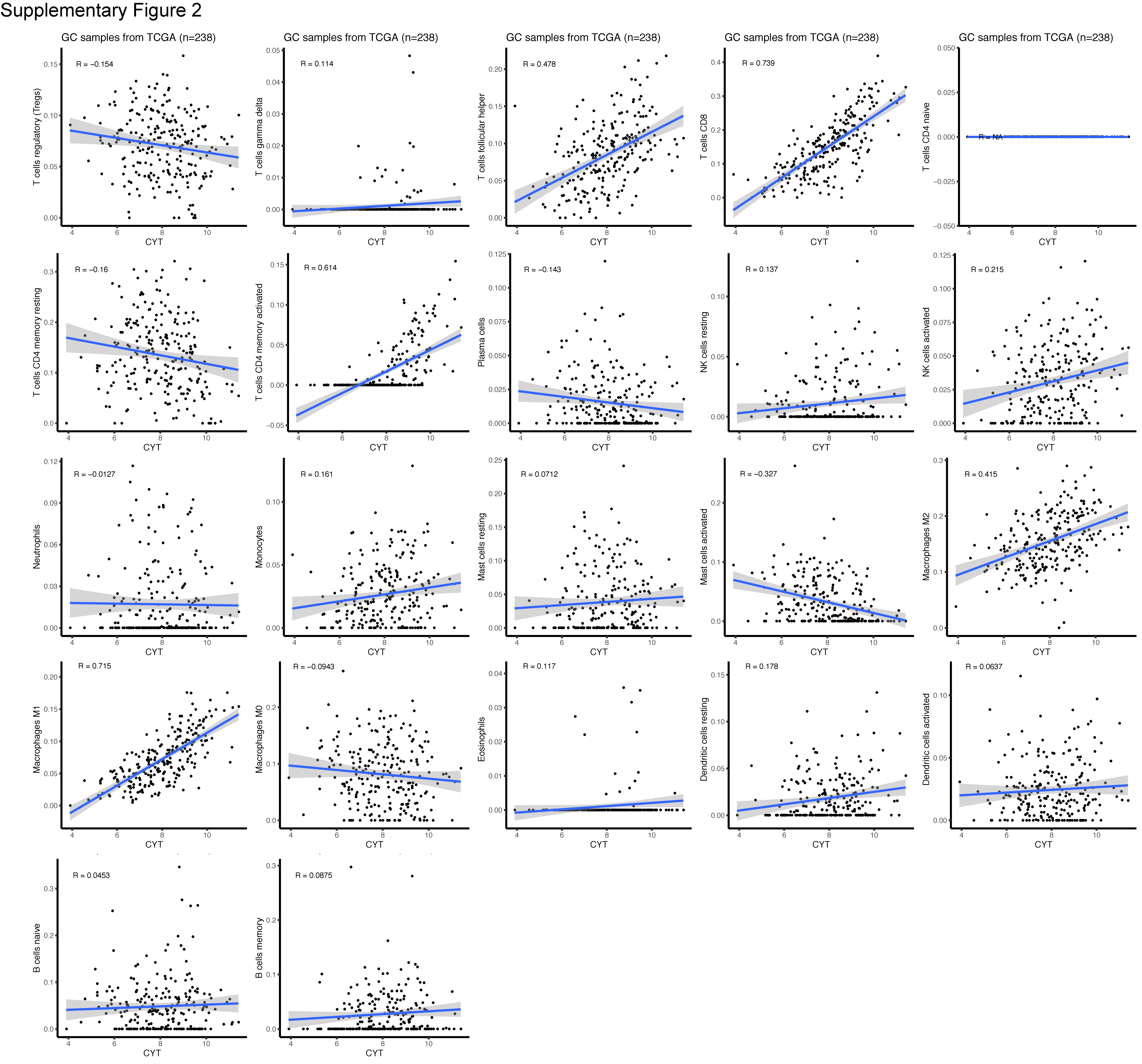

Supplement: Supplementary file 2 — Fig S2 [file CAM4-10-3129-s006.tif]

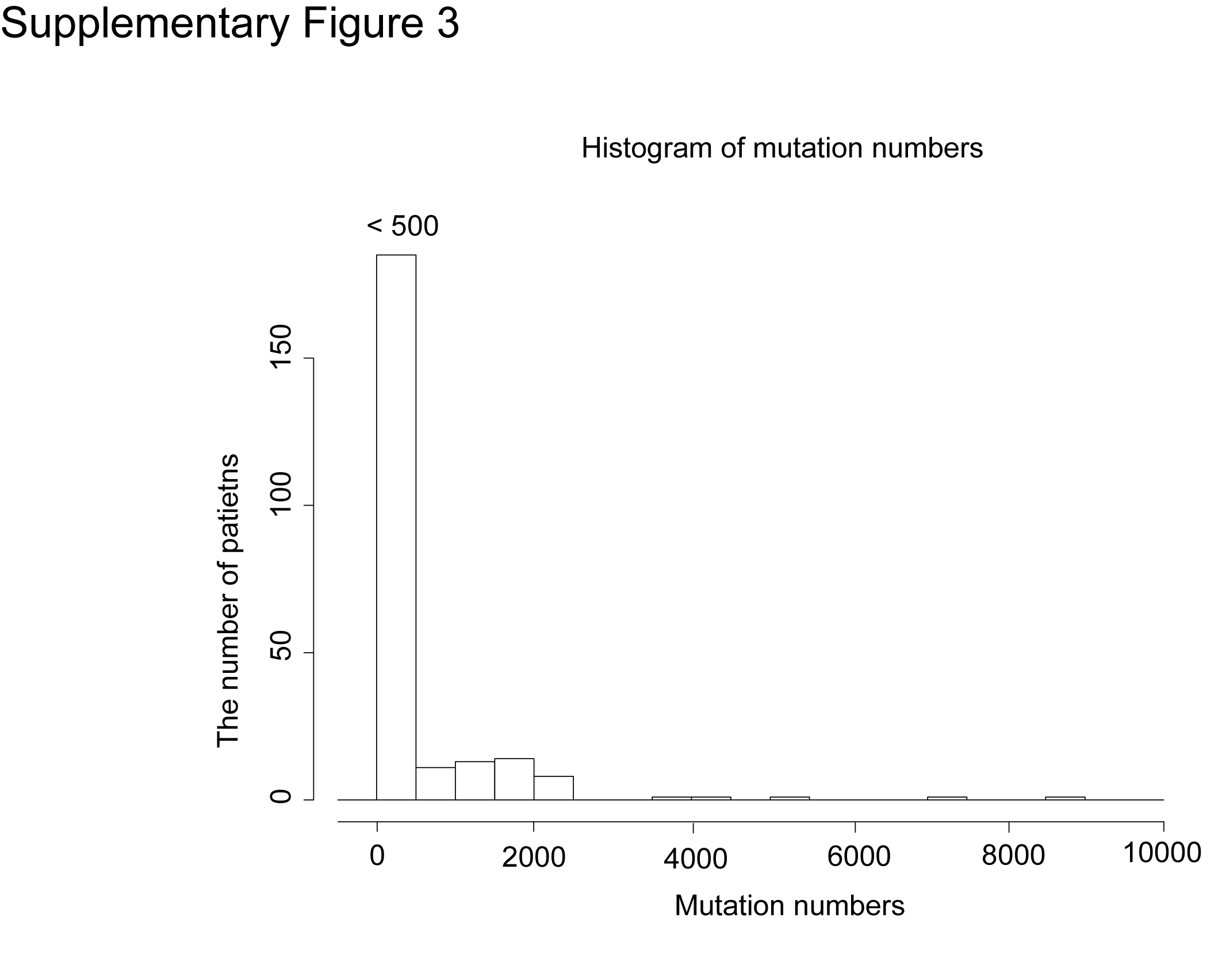

Supplement: Supplementary file 3 — Fig S3 [file CAM4-10-3129-s004.tif]

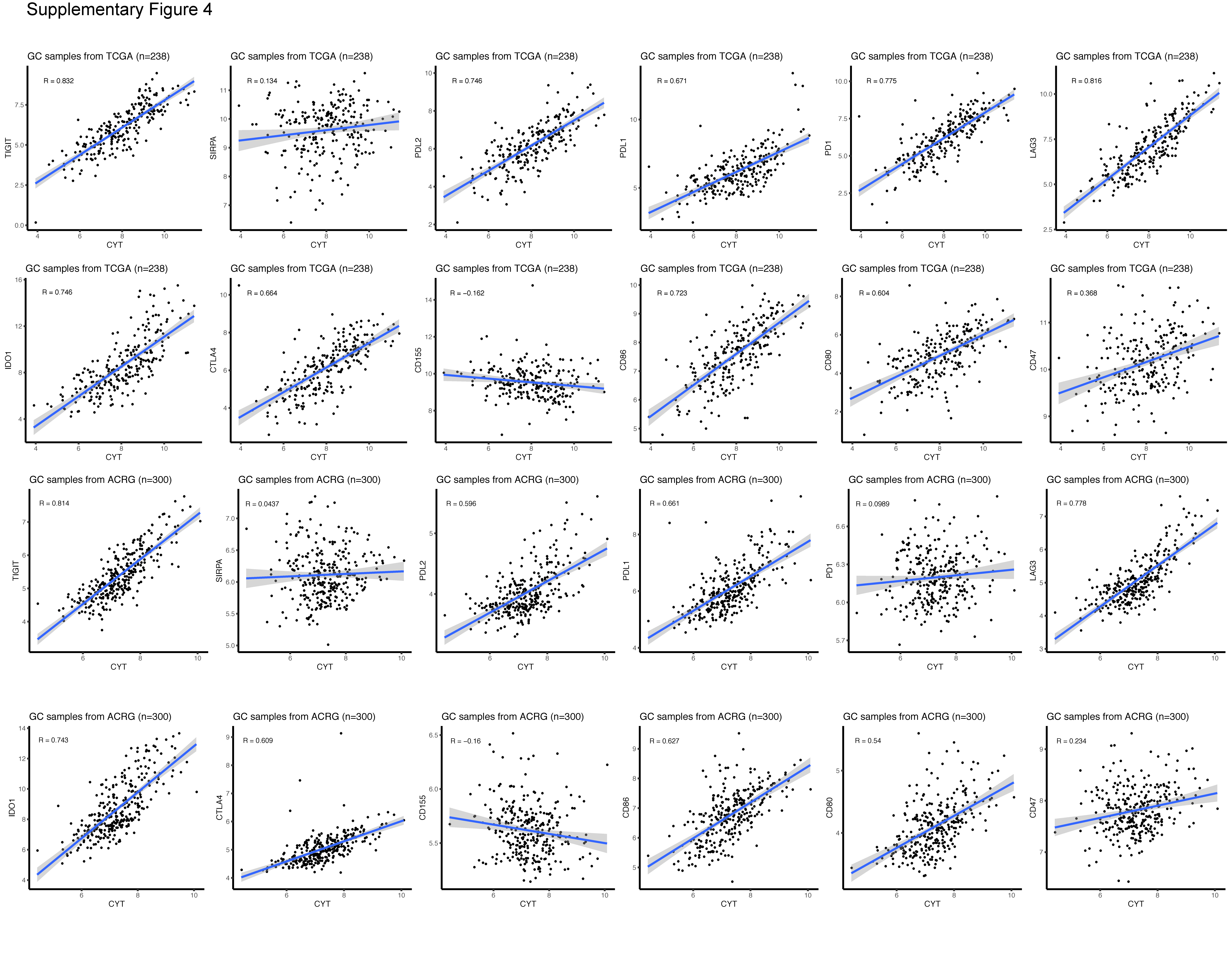

Supplement: Supplementary file 4 — Fig S4 [file CAM4-10-3129-s001.tif]

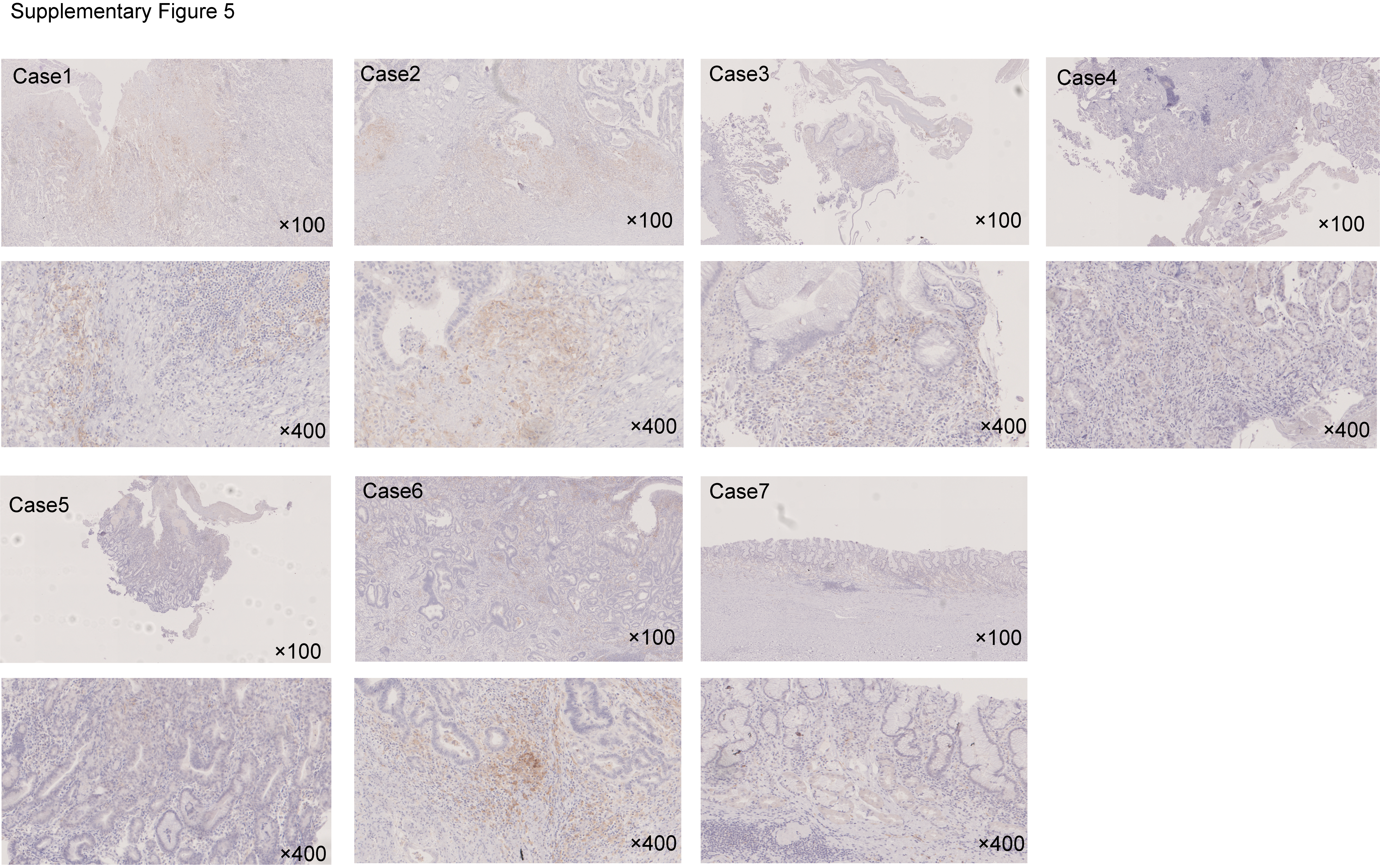

Supplement: Supplementary file 5 — Fig S5 [file CAM4-10-3129-s003.tif]
