# Supplementary material for: Cytolytic activity score as a biomarker for antitumor immunity and clinical outcome in patients with gastric cancer
Source: Cancer Med. 2021 Mar 26;10(9):3129–38. doi: 10.1002/cam4.3828 (PMC8085935; doi:10.1002/cam4.3828)
Supplement: Supplementary file 6 — Table S1 [file CAM4-10-3129-s007.docx]

**Supplementary table 1.** Gastric cancer patients who received anti-PD-1 antibody therapy in the Kyushu cohort B (n = 7).

|  | Age | Gender | CPS | Histology | Microsatellite | Previous treatment | Best response |
| --- | --- | --- | --- | --- | --- | --- | --- |
| Case1 | 63 | Male | ≥5 | Por | MSI-H | SOX | PR |
| Case2 | 69 | Male | ≥5 | Well | unknown | RAM+PAC | SD |
| Case3 | 71 | Male | ≥1 | Sig | unknown | RAM+nabPTX | non-CR/  non-PD |
| Case4 | 63 | Female | <1 | Por | unknown | SOX | non-CR/  non-PD |
| Case5 | 66 | Female | <1 | Sig | unknown | RAM+nabPTX | PD |
| Case6 | 63 | Female | ≥5 | Well | unknown | RAM+nabPTX | PD |
| Case7 | 66 | Male | <1 | Por | unknown | RAM+nabPTX | PD |

CPS: combined positive score, CR: completed response, PR: partial response, SD: stable disease, PD: progressive disease, Well: well-differentiated adenocarcinoma, Por, poorly differentiated adenocarcinoma, Sig: signet ring cell adenocarcinoma, MSI-H: high frequency microsatellite instability, SOX: TS-1 + oxaliplatin, RAM: ramucirumab, PAC: paclitaxel, nabPTX: nab-paclitaxel.
