# Supplementary material for: Cytolytic activity score as a biomarker for antitumor immunity and clinical outcome in patients with gastric cancer
Source: Cancer Med. 2021 Mar 26;10(9):3129–38. doi: 10.1002/cam4.3828 (PMC8085935; doi:10.1002/cam4.3828)
Supplement: Supplementary file 7 — Supplementary Material [file CAM4-10-3129-s002.docx]

**List of Supporting Information**

**Supplementary figure legends**

**Supplementary Figure 1. Correlation matrix for all 22 immune cell proportions in GC tissues (TCGA, n = 238).**

The values in each box represented the Pearson correlation coefficient. Negative correlation was represented in red, and positive correlation was represented in blue. The darker the color, the higher the correlation was (P < 0.05). No color (white) represented no significance (P ≥ 0.05).

**Supplementary Figure 2. Correlations between CYT score and 22 immune cell proportions in GC tissues (TCGA, n = 238).**

R in each panel represented the Pearson correlation coefficient.

**Supplementary Figure 3. Histogram of total mutation numbers in GC tissues (TCGA, n = 238).**

**Supplementary Figure 4. Correlations between CYT score and immune checkpoint molecules in GC tissues from TCGA (n = 238) and ACRG (n = 300).**

R in each panel represented the Pearson correlation coefficient.

**Supplementary Figure 5. The protein level and distribution of PD-L1 in 7 GC tissues by analysis of IHC. Original magnification, ×100 (left) and ×400 (right).**

**Supplementary Table 1. Gastric cancer patients who received anti-PD-1 antibody therapy in the Kyushu cohort B (n = 7).**
